# Multi-Modal Image Fusion Based on Matrix Product State of Tensor

**DOI:** 10.3389/fnbot.2021.762252

**Published:** 2021-11-15

**Authors:** Yixiang Lu, Rui Wang, Qingwei Gao, Dong Sun, De Zhu

**Affiliations:** Anhui University, Hefei, China

**Keywords:** multi-modal, image fusion, tensor, matrix product state, singular value decomposition

## Abstract

Multi-modal image fusion integrates different images of the same scene collected by different sensors into one image, making the fused image recognizable by the computer and perceived by human vision easily. The traditional tensor decomposition is an approximate decomposition method and has been applied to image fusion. In this way, the image details may be lost in the process of fusion image reconstruction. To preserve the fine information of the images, an image fusion method based on tensor matrix product decomposition is proposed to fuse multi-modal images in this article. First, each source image is initialized into a separate third-order tensor. Then, the tensor is decomposed into a matrix product form by using singular value decomposition (SVD), and the Sigmoid function is used to fuse the features extracted in the decomposition process. Finally, the fused image is reconstructed by multiplying all the fused tensor components. Since the algorithm is based on a series of singular value decomposition, a stable closed solution can be obtained and the calculation is also simple. The experimental results show that the fusion image quality obtained by this algorithm is superior to other algorithms in both objective evaluation metrics and subjective evaluation.

## 1. Introduction

The purpose of image fusion is to synthesize multiple images of the same scene into a fusion image containing part or all information of each source image (Zhang, 2004). The fused image contains more information than each source image, thus, it is more suitable for machine processing and human visual perception. Image fusion has a wide range of applications in many fields, such as computer vision, remote sensing, medical imaging, and video surveillance (Goshtasby and Nikolov, 2007). The same type of sensors acquire information in a similar way, so the single-modal image fusion cannot provide information of the same scene from different aspects. On the contrary, multi-modal image fusion (Ma et al., 2019) realizes the complementarity of different features of the same scene through fusing the images collected by different types of sensors and generates an informative image for subsequent processing. As typical multi-modal images, infrared and visible images, CT and MRI images can provide distinctive features and complementary information, that is, infrared images can capture thermal radiation signal and visible images can capture reflected light signal; CT is mainly used for signal acquisition of sclerous tissue (e.g., bones), and MRI is mainly used for signal acquisition of soft tissue. Therefore, multi-modal image fusion has a wide range of applications in engineering practice.

To realize image fusion, many scholars have proposed a large number of fusion algorithms in recent years. In general, the fusion methods can be divided into two categories: the spatial-domain methods and the transform-domain methods. The typical methods in the first category include the weighted average method and principal component analysis (PCA) method (Yu et al., 2011) and so on. They fuse the gray values of image pixels directly. Although the direct operation on the pixels has low complexity, the fusion process is less robust to noise, and the results cannot meet the needs of the application in most cases. To overcome this shortcoming, a fusion method based on transform is proposed (Burt and Adelson, 1983; Haribabu and Bindu, 2017; Li et al., 2019). In general, the transform-based methods obtain the transformed coefficients of an image using a certain set of base functions, then fuse these coefficients through certain fusion rules, and finally obtain the final fused image through the corresponding inverse transform. For example, Burt and Adelson (1983) formed a laplacian pyramid (LP) by desampling and filtering source images, and then designed different fusion strategies at each layer. Finally, the fused image is obtained by applying the inverse transform on the fusion coefficients. Haribabu and Bindu (2017) first decomposed the source images by using discrete wavelet transform (DWT) and fused the coefficients with predefined fusion rules, and then obtained the final image by applying the inverse discrete wavelet transform on fused coefficients. Because the transform-based method employs the average weighted fusion rules for the low-frequency components which carry the most energy of the image, there will be something wrong with the contrast loss of the final fused image.

In addition to traditional spatial-domain and transform-domain methods, sparse representation (SR) has been extensively used in image fusion in recent years (Yang and Li, 2010; Jiang and Wang, 2014; Liu et al., 2016; Zhang and Levine, 2016). The SR method assumes that the signal to be processed satisfies *y* ∈ *R*^*n*^, then *y* = *Dx*, where *D* ∈ *R*^*n*×*m*^(*n* << *m*) is an overcomplete dictionary, and *n* is the dimensions of the signal and *m* is the number of atoms in the dictionary *D* which is formed by a set of image subblocks, *x* is the sparse coefficients vector. The fused image is reconstructed by means of fusing the sparse coefficients. Although the SR-based method has achieved many results in the field of image fusion, some detailed information will be lost in the reconstructed image (e.g., the edges and textures tend to be smoothed), which limits the ability of the SR to express images (Yang and Li, 2010). To solve this problem, some scholars proposed some improved algorithms (Jiang and Wang, 2014; Liu et al., 2016). For instance, Jiang and Wang (2014) used morphological component analysis (MCA) to represent the source images more effectively. The MCA method first applied SR to separate the source images into two parts: cartoon and texture, then different fusion rules were designed to fuse these two parts respectively. Finally, a fused image with rich information was obtained, and more characteristic features of the source images were preserved.

As an extension of the vector and matrix, the tensor (Kolda and Bader, 2009) plays an important role in the high-dimensional data processing. In the field of computer science and technology, a tensor is a multi-dimensional array. It can be extended to some common data types, for example, a zero-order tensor can be defined as a constant, the tensor of order 1 is defined as a vector, the tensor of order 2 is defined as a matrix, the tensor of order 3 and the tensor of order *N* (*N* ≥ 3) is called high-order tensor. In essence, tensor decomposition is a high-order generalization of matrix decomposition, which is mainly applied to dimensionality reduction, sparse data filling, and implicit relationship mining. The information processing method based on tensor is more suitable for the processing of high-dimensional data and the extraction of feature information than vector and matrix, therefore, some relevant applications have been emerged in recent years (Bengua et al., 2015, 2017a,b; Zhang et al., 2016). In view of the excellent performance of tensors in representing high-dimensional data and feature extraction, a tensor-based high-order singular value decomposition method (HOSVD) (Liang et al., 2012) was applied to image fusion and achieved good results. In this method, the source image is initialized into a tensor which is subsequently decomposed into several sub-tensors by using a sliding window technique. Then, the HOSVD is applied on each sub-tensors to extract the corresponding features which are fused by employing certain fusion rules.

Since HOSVD is an approximate decomposition method, it will lead to the loss of information in the process of image fusion. At the same time, the calculation process is large and a stable closed-form solution cannot be obtained. To avoid loss of detailed information, a novel method based on matrix product state (MPS) is proposed to fuse the multi-modal images. Compared with HOSVD, MPS achieves the improvement of HOSVD and achieves the purpose of acquisition image information accurately. Moreover, being different from SR who linearly represents images by using atoms in an overcomplete dictionary, MPS decomposes image tensor into MPS. The main difference is that SR is approximate decomposition, while MPS is accurate decomposition. Therefore, in terms of signal reconstruction, MPS has better performance in signal expression. The main contributions of the article are outlined as follows: (i) Considering that image fusion depends more on local information of the source images and dividing the image into blocks can get more details of each pixel, the two source images are first divided into several sub-image blocks, and then the corresponding sub-image blocks are initialized into sub-tensors; (ii) We perform the MPS on each sub-tensor separately to obtain the corresponding core matrixes. The core matrixes are fused using the fusion rule based on the sigmoid function which incorporates the conventional choose-max strategy and the weighted average strategy. This fusion strategy can preserve the features of the multi-modal source images and reduce the loss of contrast to the greatest extent; (iii) Due to the application of MPS, the computational complexity of image fusion based on tensor is reduced dramatically. Hence, MPS decomposition is realized by computing a series of sub-tensors with maximum order 3. Moreover, a stable closed-form solution can also be obtained in the proposed algorithm.

The rest of the article is organized as follows. Section 2 introduces the theory of matrix product decomposition. In section 3, the algorithm principle and the fusion steps are detailly discussed. Subsequently, the results of the experiments are presented in section 4. Finally, some conclusions are drawn in section 5.

## 2. MPS for Tensor

### 2.1. Tensor

Tensor is a generalization of the vector. A vector is a kind of tensor with order 1. For simplicity and accuracy of the following expressions, first, we introduce some notations about tensors. The tensor of order 0 is a constant, represented by lowercase letter *x*; the tensor of order 1 is a vector represented by a bold lowercase letter **x**; the tensor of order 2 is a matrix represented by a bold capital letter **X**; the tensor of order 3 is a tensor represented by bold capital letters in italics ***X***. In this way, a tensor of order N and the size of each dimension are *I*_1_ × *I*_2_ × ⋯ × *I*_*N*_ can be expressed as $X\in R^{I_{1}\times I_{2}\times \cdots \times I_{N}}$, where *I*_*i*_ corresponds to the length of the *i*-th dimension. In general, we use *x*_*i*_1__ ⋯ *x*_*i*_*N*__ to represent the (*i*_1_, ⋯ , *i*_*N*_)-th element of ***X***.

### 2.2. MPS for Tensor

**Figure 1:**
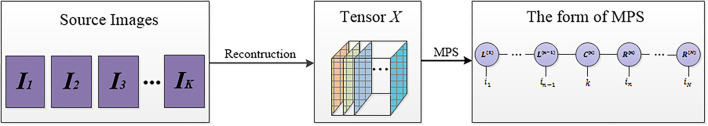
The matrix product state (MPS) form of ***X***.

The MPS decomposition (Perez-Garcia et al., 2006; Schollwock, 2011; Schuch et al., 2011; Sanz et al., 2016) aims to decompose an N-dimensional tensor ***X*** into the corresponding left-right orthogonal factor matrix and a core matrix. First, all the dimensions of an N-dimensional tensor ***X*** are rearranged, which lets the dimension K corresponding to the number of images to be fused, for example, if the number of source images is equal to 2, then *K* = 2. Additionally, the tensor ***X*** satisfies $X\in R^{I_{1}\times \cdots \times I_{n-1}\times K\times I_{n}\times \cdots \times I_{N}}$, *I*_1_ ≥ ⋯ ≥ *I*_*n*−1_, *I*_*n*_ ≤ ⋯ ≤ *I*_*N*_, then the elements in the tensor ***X*** can be expressed in the form of MPS, and the schematic diagram of MPS form of ***X*** is shown in Figure 1:

(1) $$\begin{array}{r} x_{i_{1}\cdots k\cdots i_{N}}=x_{i_{1}\cdots i_{n}\cdots i_{N}}^{\left(k\right)}\approx {\text{L}}_{i_{1}}^{\left(1\right)}\cdots {\text{L}}_{i_{n-1}}^{\left(n-1\right)}{\text{C}}_{k}^{\left(n\right)}{\text{R}}_{i_{n}}^{\left(n+1\right)}\cdots {\text{R}}_{i_{N}}^{\left(N+1\right)}. \end{array}$$

${\text{L}}_{i_{j}}^{\left(j\right)}$ and ${\text{R}}_{i_{\left(j-1\right)}}^{\left(j\right)}$ mentioned in the above formula are called left-right orthogonal factor matrix with size δ_*j*−1_ × δ_*j*_, where δ_0_ = δ_*N*+1_ = 1, and they are all orthogonal:

(2) $$\begin{array}{r} \overset{I_{j}}{\underset{i_{j}=1}{\sum }}(L_{i_{j}}^{\left(j\right)}{)}^{T}{\text{L}}_{i_{j}}^{\left(j\right)}=\text{I},\;\left(j=1,\cdots \;,n-1\right) \end{array}$$

and

(3) $$\begin{array}{r} \overset{I_{j-1}}{\underset{i_{j-1}=1}{\sum }}{\text{R}}_{i_{j-1}}^{\left(j\right)}(R_{i_{j-1}}^{\left(j\right)}{)}^{T}=\text{I},\;\left(j=n+1,\cdots \;,N+1\right), \end{array}$$

where **I** is an identity matrix, ${\text{C}}_{k}^{n}$ is called core matrix.

A tensor ***X*** can be decomposed into the form of (1) through two series of SVD decomposition. The process includes a left-to-right sweep and a right-to-left sweep. We summarize it in Algorithm 1.

**Algorithm 1:** Feature Extraction based on MPS

**Input:**
$X\in R^{I_{1}\times \cdots \times I_{n-1}\times K\times \cdots \times I_{N}}$
**Main Procedure:**
1: Set ${\text{W}}^{\left(1\right)}={\text{X}}_{\left(1\right)}$;
2: **for** *j* = 0, 1, …, *n* − 1 **do**
3: **W**^(*j*)^ = **USV**;
4: Reshape **U**^(*j*)^ to***U***;
5: ${\text{L}}_{i_{j}}^{\left(j\right)}=U\left(:,i_{j},:\right)$;
6: **end for**
7: Reshape **V**^(*n*−1)^ to${\text{W}}^{N}\in R^{\left(\Delta_{n-1}K\cdots I_{N}\right)\times I_{N}}$;
8: **for** *j* = *N, N* − 1, …, *n* **do**
9: **W**^(*j*)^ = **USV**;
10: Reshape **V**^(*j*)^ to ***V***;
11: ${\text{R}}_{i_{j-1}}^{\left(j+1\right)}=V\left(:,i_{j-1},:\right)$;
12: **end for**
13: Reshape **U**^(*n*)^into $C\in R^{\left(I_{n-1}K\cdots I_{N}\right)\times I_{N}}$;
14: Set ${\text{C}}_{k}^{n}=C\left(:,k,:\right)$.
**Output:**
Core Matrix: ${\text{C}}_{k}^{n}\in R^{\Delta_{n-1}\times \Delta_{n}},k=1,\cdots \;,K$;
Left Factor Matrix: ${\text{L}}_{i_{j}}^{\left(j\right)}\left(i_{j}=1,\cdots \;,I_{j},j=1,\cdots \;,n-1\right)$;
Right Factor Matrix: ${\text{R}}_{i_{\left(j-1\right)}}^{\left(j\right)}\left(i_{\left(j-1\right)}=1,\cdots \;,I_{\left(j-1\right)},j=n+1,\cdots \;,N+1\right)$

## 3. Image Fusion Based on MPS

**Figure 2:**
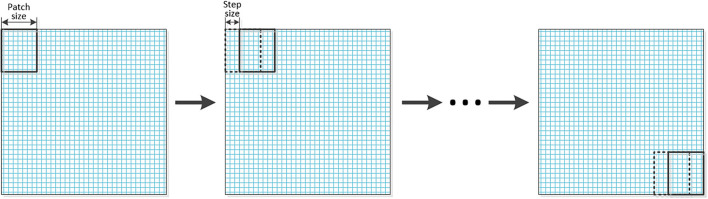
The graphical representation of the sliding window technology.

In this section, the whole process of image fusion will be described. The source images which have been reconstructed into tensors are decomposed into a series of sub-tensors by using the sliding window technology. The graphical representation of the sliding window technology is shown in Figure 2. Then MPS is applied to the decomposed sub-tensors to obtain the core matrixes, and the sigmoid function is used for the fusion of each pair of core matrixes to obtain the fused core matrixes.

The specific theoretical concepts of decomposition and fusion are described in sections 3.1, 3.2, respectively, and the overall process of image fusion proposed in this article is described in section 3.3.

### 3.1. Tensor Decomposition by MPS

For the two source images A and B with sizes of *M* × *N*, we use them to construct a tensor X with dimension *M* × *N* × 2. Taking into account the importance of local information of the source image, a sliding window technology is used to decompose it into several sub-tensors ***F*** with dimension $\bar{M}\times \bar{N}\times 2$, and the sliding step *p* used should satisfy $p\leq \min\left\{\bar{M},\bar{N}\right\}$; the sub-tensor ***F*** is obtained by the Algorithm 2, as follows. In Algorithm 2, the fix((*M* − *patch size*)/*stepsize*) represents the nearest integer to (*M* − *patch size*)/*stepsize* and fix((*N* − *patch size*)/*stepsize*) represents the nearest integer to (*N* − *patch size*)/*stepsize*. Then, MPS is applied to each of the sub-tensors.

**Algorithm 2:** The Sub-tensor obtained by Sliding Window Technology

**Input:**
**X** ∈ *R*^*M* × *N* × 2^
**Main Procedure:**
1: **for** $i=1,1+stepsize,\ldots ,1+stepsize\times fix\left(\frac{M-patch\;size}{stepsize}\right)$ **do**
2: **for** $j=1,1+stepsize,\ldots ,1+stepsize\times fix\left(\frac{N-patch\;size}{stepsize}\right)$ **do**
3: **F** = **X**(*i*:*i* + *patch size* − 1, *j* + *patch size* − 1, :);
4: **end for**
5: **end for**
**Output:**
sub-tensor: $\text{F}\in R^{\bar{M}\times \bar{N}\times 2}$;

### 3.2. Design of Fusion Rule

We introduce the sigmoid function as the fusion rule of the characteristic coefficients, the fusion coefficient of each core matrix can be defined as follows:

(4) $$\begin{array}{r} e_{i}\left(l\right)=\overset{\bar{M}}{\underset{m=1}{\sum }}\overset{\bar{N}}{\underset{n=1}{\sum }}|{\text{C}}_{i}\left(m,n,l\right)|\;l=1,2 \end{array}$$

where the subscript *i* indicates the number of each sub-image, and *l* is the label of the corresponding source image.

For *e*_*i*_(*l*) obtained in the previous section, the fusion rule is selected by comparing the values of *e*_*i*_(1) and *e*_*i*_(2). When *e*_*i*_(1) is much less or much more than *e*_*i*_(2), we use the Max rule, and when the relationship between *e*_*i*_(1) and *e*_*i*_(2) satisfy the other relation, we use weighted fusion to fuse the corresponding coefficient matrix and then get the final fusion coefficient matrix. The function is as follows:

(5) $$\begin{array}{l} D_{i}=\frac{1}{1+exp\left(-kln\left(\frac{e_{i}\left(1\right)}{e_{i}\left(2\right)}\right)\right)}\\ \;\times {\text{C}}_{i}\left(:,:,1\right)+\frac{exp\left(-kln\left(\frac{e_{i}\left(1\right)}{e_{i}\left(2\right)}\right)\right)}{1+exp\left(-kln\left(\frac{e_{i}\left(1\right)}{e_{i}\left(2\right)}\right)\right)}\times {\text{C}}_{i}\left(:,:,2\right) \end{array}$$

where *k* is the shrinkage factor of the mentioned sigmoid function. After **D**_*i*_ is obtained, each of the fused sub-image blocks *F*_*i*_ can be reconstructed by the inverse operations of MPS. Then the sub-image blocks *F*_*i*_ is used to obtain the final fused image G.

**Figure 3:**
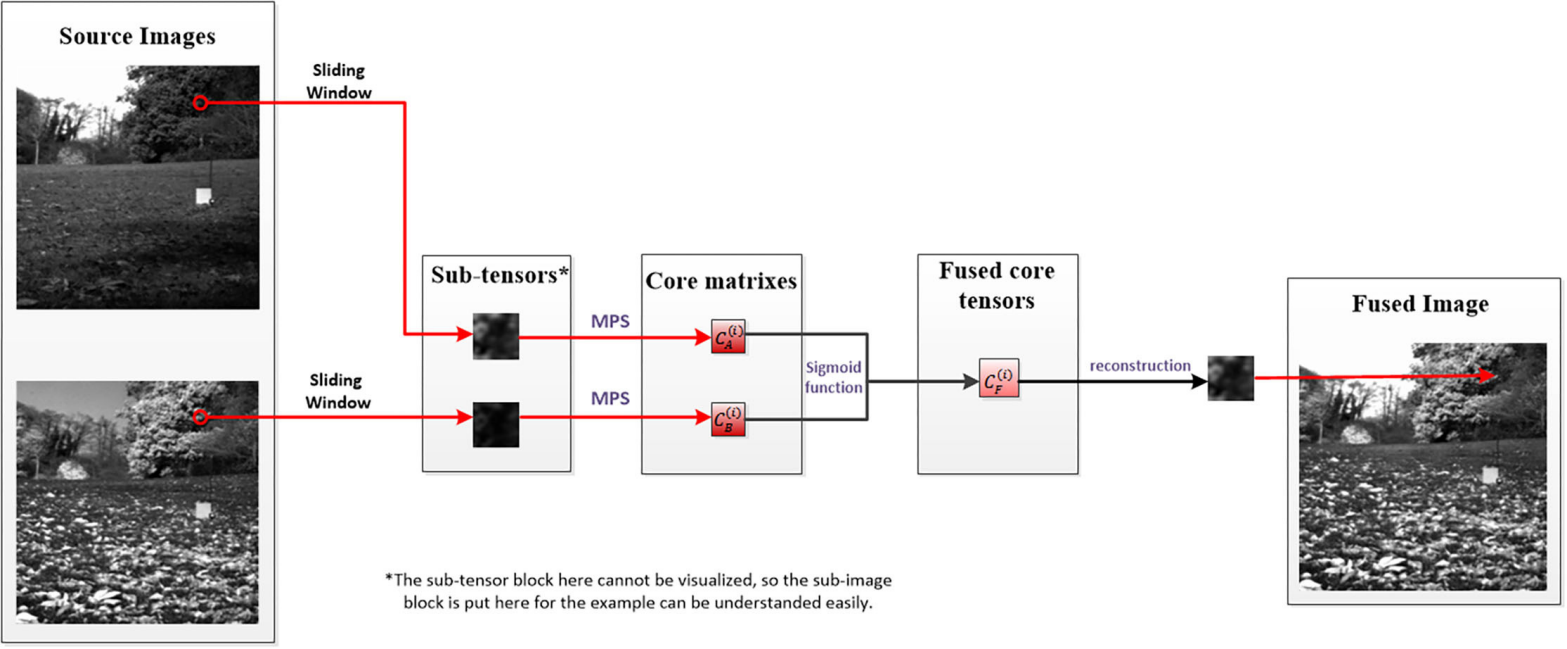
An example for visualizing the process of decomposition and fusion.

To make the process of decomposition and fusion more concrete, the first group of the experiment images is used as an example to make a flowchart as shown in Figure 3:

### 3.3. The Process of Image Fusion Based on MPS

The process of image fusion based on MPS can be divided into the seven steps as follows

1. Input two source images;
2. Reconstructed the two source images into a third-order tensor, and the sub-tensors are extracted by sliding window technology;
3. Matrix product state decomposition is used on sub-tensors to obtain left and right factor matrixes and core matrixes;
4. Compare the vectors representing source image 1 and source image 2 in the core matrixes obtained in step 3, and obtain the fused matrixes by corresponding their quantitative relations to different situations of the sigmoid function, and then construct it as sub-tensors;
5. Multiply the fused sub-tensors by left and right factor tensors to obtain sub-images;
6. Sub-images addition;
7. Output fused image.

**Figure 4:**
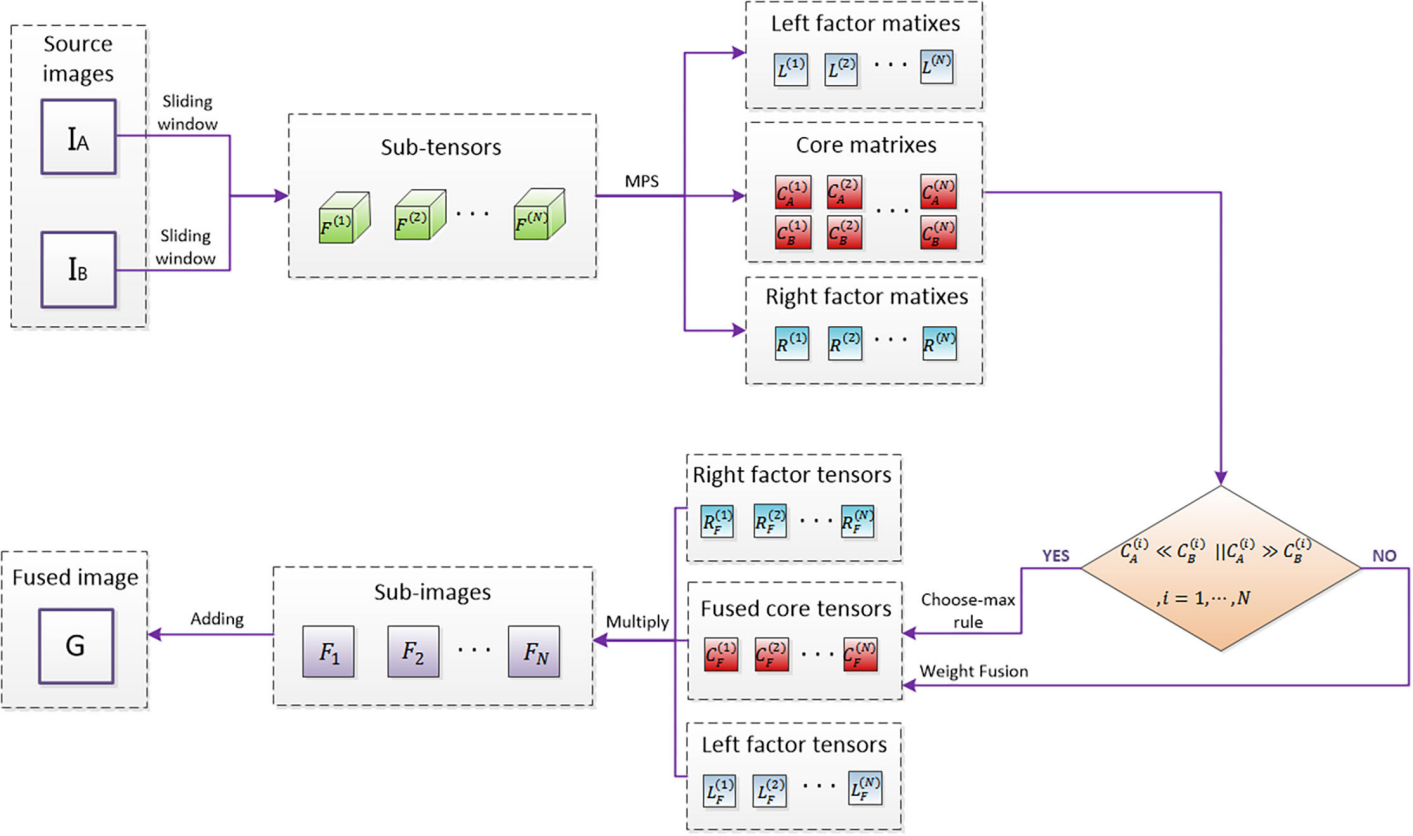
Fusion flowchart based on MPS.

The specific flowchart is shown in Figure 4.

## 4. Experiments

### 4.1. Objective Evaluation Metrics

1. Standard deviation (SD)
  SD is defined as follows:
  (6) $$\begin{array}{r} SD=\sqrt{\frac{1}{H\times W}\overset{H}{\underset{x=1}{\sum }}\overset{W}{\underset{y=1}{\sum }}{\left(F\left(x,y\right)-\mu \right)}^{2}}, \end{array}$$
  where μ is the average value of the fused image, *H* and *W* are the length and width of the image, respectively. *SD* is mainly used to measure the contrast of the fused image.
2. Mutual information (MI)
  Mutual information is defined as follows:
  (7) $$\begin{array}{r} MI=\overset{L}{\underset{x=1}{\sum }}\overset{L}{\underset{y=1}{\sum }}h_{R,F}\left(i,j\right)log_{2}\frac{h_{R,F}\left(i,j\right)}{h_{R}\left(i\right)+h_{F}\left(j\right)}, \end{array}$$
  where *h*_*R,F*_(*i, j*) is the normalized joint distribution gray histogram between the source image R and the fused image F, *h*_*R*_(*i*) and *h*_*F*_(*j*) are the normalized marginal distribution histogram of the two source images, respectively, *L* is the number of gray levels.
3. Structural similarity (SSIM)
  Structural similarity is defined as follows:
  (8) $$\begin{array}{r} SSIM\left(x,y\right)={\left(\frac{2\mu_{x}\mu_{y}+c_{1}}{\mu_{x}^{2}+\mu_{y}^{2}+1}\right)}^{\alpha }{\left(\frac{2\sigma_{x}\sigma_{y}+c_{1}}{\sigma_{x}^{2}+\sigma_{y}^{2}+1}\right)}^{\beta }{\left(\frac{\sigma_{xy}+c_{3}}{\sigma_{x}\sigma_{y}+c_{3}}\right)}^{\gamma }, \end{array}$$
  where μ_*x*_ and μ_*y*_ are the average value of *x* and *y*. The middle term represents the similarity of contrast, σ_*x*_ and σ_*y*_ is the SD of *x* and *y*. The right term characterizes the structural similarity, and σ_*xy*_ is the covariance of *x* and *y*. The *c*_1_, *c*_2_, and *c*_3_ are three constants, and the parameters α, β, and γ, respectively, adjust the contribution of the three terms. *SSIM* can calculate the similarity between the fused image and the source image. Its value which is between 0 and 1 is closer to 1, the more similar the two images are. The average value of the fused image and the two source images A and B is taken as the final evaluation metric, namely
  (9) $$\begin{array}{r} SSIM=\frac{1}{2}\left(SSIM_{A}+SSIM_{B}\right). \end{array}$$
4. Gradient based fusion metric (*Q*_*G*_)
  *Q*_*G*_ is defined as follows:
  (10) $$\begin{array}{r} Q_{G}=\frac{\sum_{x=1}^{H}\sum_{y=1}^{W}\left(Q_{AF}\left(x,y\right)w_{A}\left(x,y\right)+Q_{BF}\left(x,y\right)w_{B}\left(x,y\right)\right)}{\sum_{x=1}^{H}\sum_{y=1}^{W}\left(w_{A}\left(x,y\right)+w_{B}\left(x,y\right)\right)}, \end{array}$$
  where *Q*_*AF*_(*x, y*) = *Q*_*AF*_*g*__(*x, y*)*Q*_*AF*_α__(*x, y*), at each pixel (*x, y*),*Q*_*AF*_*g*__(*x, y*) and *Q*_*AF*_α__(*x, y*) denote the edge strength and orientation preservation values. *Q*_*BF*_(*x, y*) is defined as the same as *Q*_*AF*_(*x, y*). The weighting factors *w*_*A*_(*x, y*) and *w*_*B*_(*x, y*) indicate the significance of *Q*_*AF*_(*x, y*) and *Q*_*BF*_(*x, y*). *Q*_*G*_ is an important fusion image quality evaluation method computing the amount of gradient information that is injected into the fused image from the source image.
5. Phase congruency based fusion metric (*Q*_*P*_)
  The *Q*_*P*_ is defined as follows:
  (11) $$\begin{array}{r} Q_{P}={\left(P_{p}\right)}^{\alpha }{\left(P_{M}\right)}^{\beta }{\left(P_{m}\right)}^{\gamma }, \end{array}$$
  where *p*, *M*, and *m* refer to phase congruency, maximum, and minimum moments. The parameters α, β, and γ are set to 1 in this article. For more detailed information on parameters, please refer to the article Hong (2000). *Q*_*P*_ measures the extent that the salient features in the source image are preserved.

### 4.2. Study of Patch Size and Step Size

**Figure 5:**
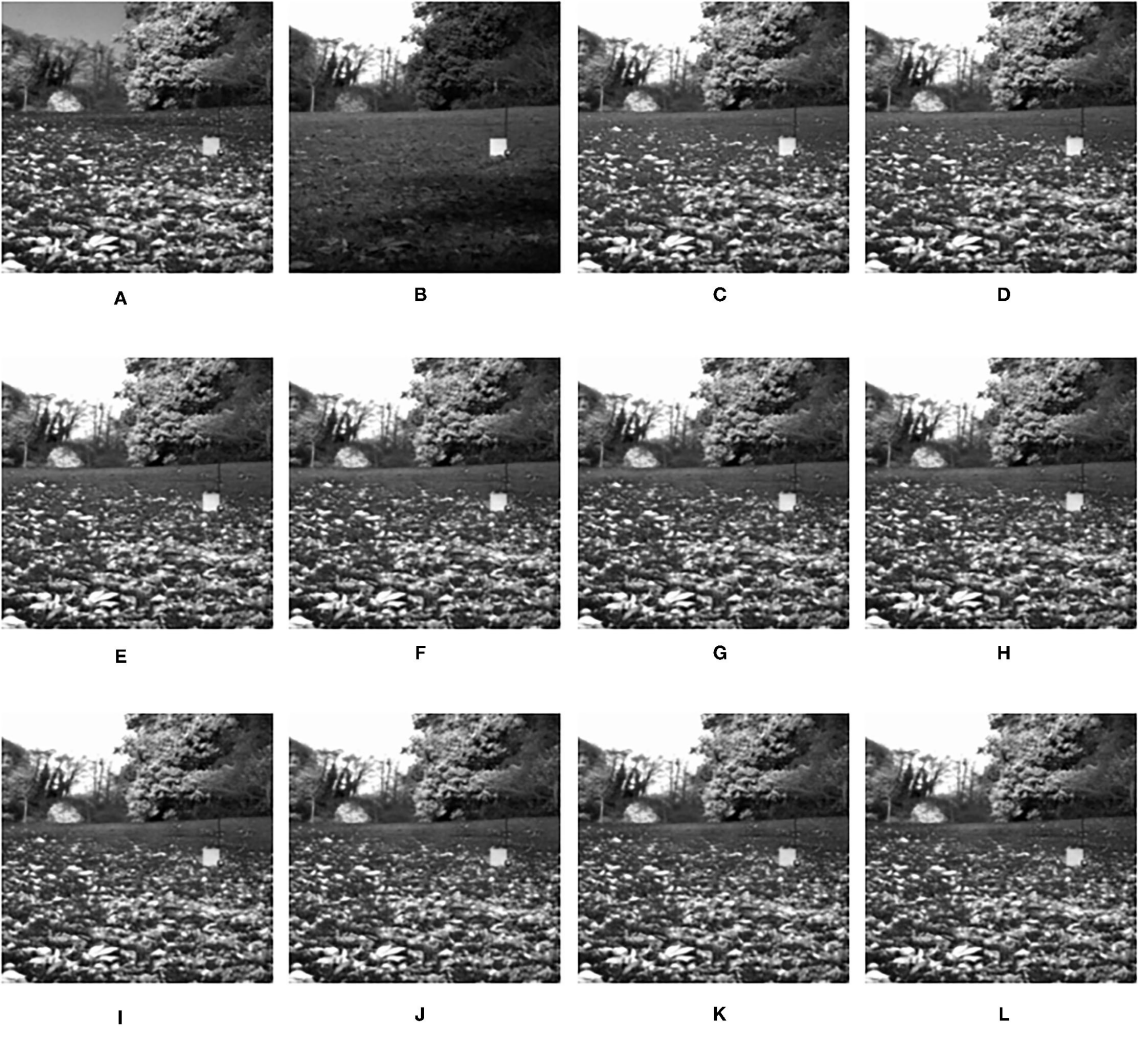
The output fused images in patch size experiment. **(A)** original image (infrared image); **(B)** original image (visible image); **(C)** patch of size 2 × 2; **(D)** patch of size 4 × 4; **(E)** patch of size 6 × 6; **(F)** patch of size 8 × 8; **(G)** patch of size 10 × 10; **(H)** patch of size 12 × 12; **(I)** patch of size 14 × 14; **(J)** patch of size 16 × 16; **(K)** patch of size 18 × 18; **(L)** patch of size 20 × 20.

**Figure 6:**
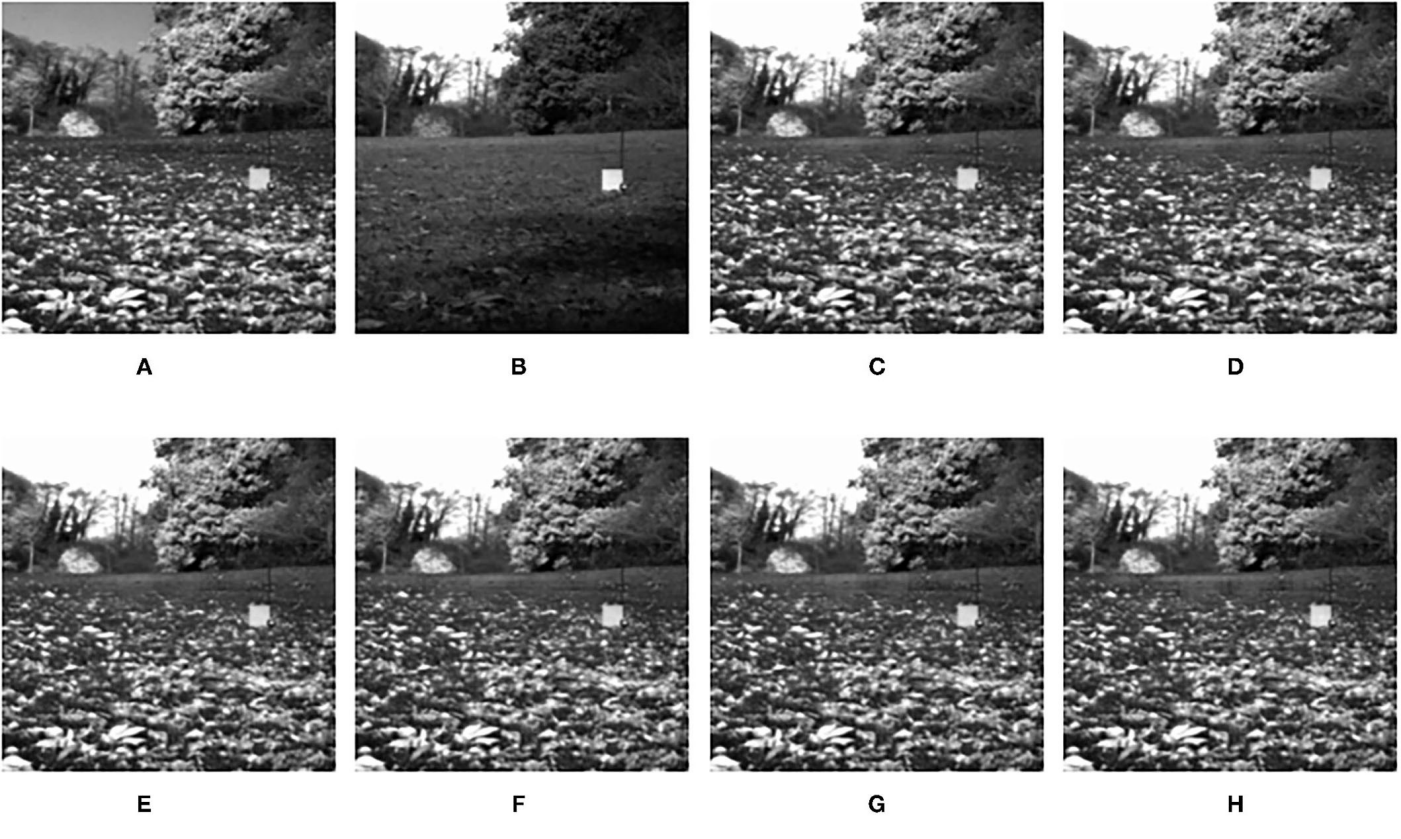
The output fused images in step size experiment. **(A)** original image (infrared image); **(B)** original image (visible image); **(C)** step size = 1; **(D)** step size = 2; **(E)** step size = 4; **(F)** step size = 6; **(G)** step size = 8; **(H)** step size = 10.

Considering the sliding window technology is used, we will first study the respective influence of the size of the sub-image block and the step size of the sliding window on the performance of the fusion image experimentally. In the following statement we use patch size and step size to call the two factors briefly. To obtain the optimal patch size and step size, we will use a pair of infrared and visible images as source images, as shown in Figures 5A,B. In the experiment of patch size, the patch size is set to 2 × 2, 4 × 4, 6 × 6, 8 × 8, 10 × 10, 12 × 12, 14 × 14, 16 × 16, 18 × 18, and 20 × 20 with the step size fixed to 1 and shrinkage factor fixed to 200. In the experiment of step size, the step size is set to 1, 2, 4, 6, 8, and 10 with the patch size fixed to 16 × 16 and the shrinkage factor fixed to 200. The experimental results based on the objective evaluation metrics are shown in Tables 1, 2. The output fused images are shown in Figures 5, 6.

**Table 1:** The influence of patch size.

**Patch size**	**SD**	**MI**	**SSIM**	***Q*_*G*_**	***Q*_*P*_**
2 × 2	63.9385	**0.9289**	0.6683	0.6543	0.7358
4 × 4	64.3397	0.8837	0.6680	0.6589	0.7677
6 × 6	64.5772	0.8810	0.6679	0.6658	0.7819
8 × 8	64.7225	0.8850	0.6678	0.6710	0.7893
10 × 10	64.8229	0.8835	0.6681	0.6738	0.7903
12 × 12	64.9079	0.8867	0.6686	0.6760	0.7900
14 × 14	64.9586	0.8846	0.6695	0.6783	0.7907
16 × 16	65.0244	0.8811	**0.6699**	**0.6813**	**0.7915**
18 × 18	66.0479	0.8765	0.6543	0.6663	0.7574
20 × 20	**66.1362**	0.9043	0.6532	0.6679	0.7573

*Bold values mean maximum value of the same metrices in the same group of comparative experiments*.

**Table 2:** The influence of step size.

**Step size**	**SD**	**MI**	**SSIM**	***Q*_*G*_**	***Q*_*P*_**
1	65.0244	0.8811	**0.6699**	**0.6813**	**0.7915**
2	65.0206	0.8832	0.6697	0.6809	0.7910
4	65.0283	0.8905	0.6690	0.6775	0.7888
6	**65.0316**	0.9087	0.6673	0.6751	0.7835
8	65.0304	0.9223	0.6666	0.6753	0.7811
10	64.1665	**0.9461**	0.6630	0.6778	0.7752

*Bold values mean maximum value of the same metrices in the same group of comparative experiments*.

It can be seen clearly from Table 1, in most cases, that the best results can be obtained when the size of the sub-image block is 16 × 16. According to simple analysis, when the sub-image block is too small, the image characteristics cannot be effectively represented. Additionally, it can be seen from Table 2 that when the step size is 1, the best result can be obtained. According to simple analysis, when the step size is too large, local information of the image may be lost or cannot be displayed well. Therefore, the in following experiments, the patch size was set to 16 × 16, and the step size was set to 1.

### 4.3. Computation Complexity

The computation time of each group of experimental images is recorded when different fusion algorithms are used. Experimental results show that the complexity of the proposed algorithm is lower than other algorithms. The results are shown in Table 3 as follows:

**Table 3:** Computation times of different algorithms.

**Methods**	**Times (s)**
*DWT*	0.1796
*LP*	0.3812
*SR*	6.4527
*DTCWT*−*SR*	3.8822
*VGG*	2.5067
*MPS*	1.8357

All the codes are performed under MATLAB R2014a running on computer equipment with an Intel i7-7700K CPU (4.2 GHz) and 16 GB of RAM. As can be seen from the table, compared with SR and Dual-tree complex wavelet transform-sparse representation (DTCWT-SR), the running of the proposed algorithm is faster. In general, the computational complexity of the proposed algorithm is reduced.

### 4.4. Experimental Results and Discussion

**Figure 7:**
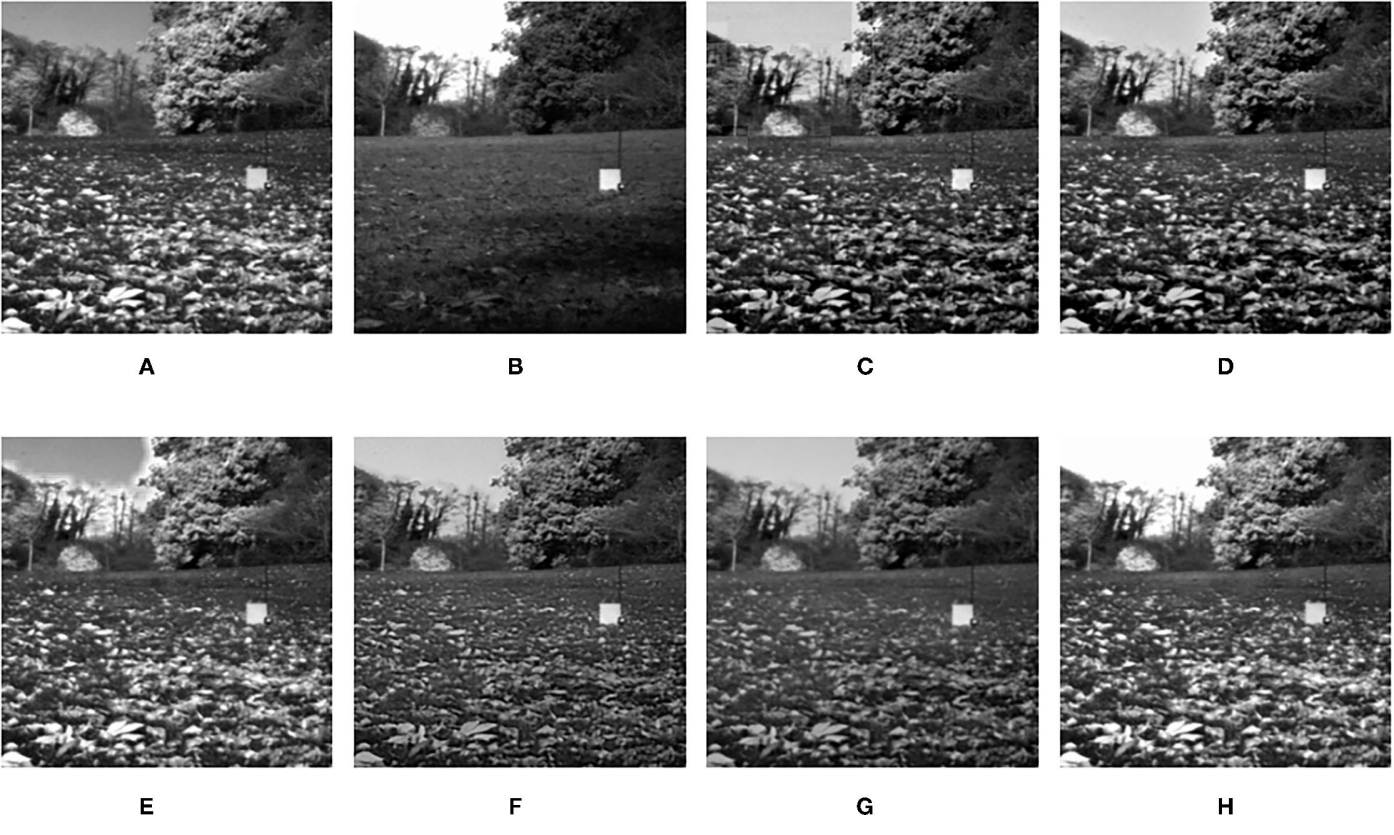
Comparison experimental results of infrared and visible images. **(A)** original figure (infrared image); **(B)** original figure (visible image); **(C)** discrete wavelet transform (DWT); **(D)** laplacian pyramid (LP); **(E)** sparse representation (SR); **(F)** Dual-tree complex wavelet transform-sparse representation (DTCWT-SR); **(G)** VGG; **(H)** Matrix product state (MPS).

**Figure 8:**
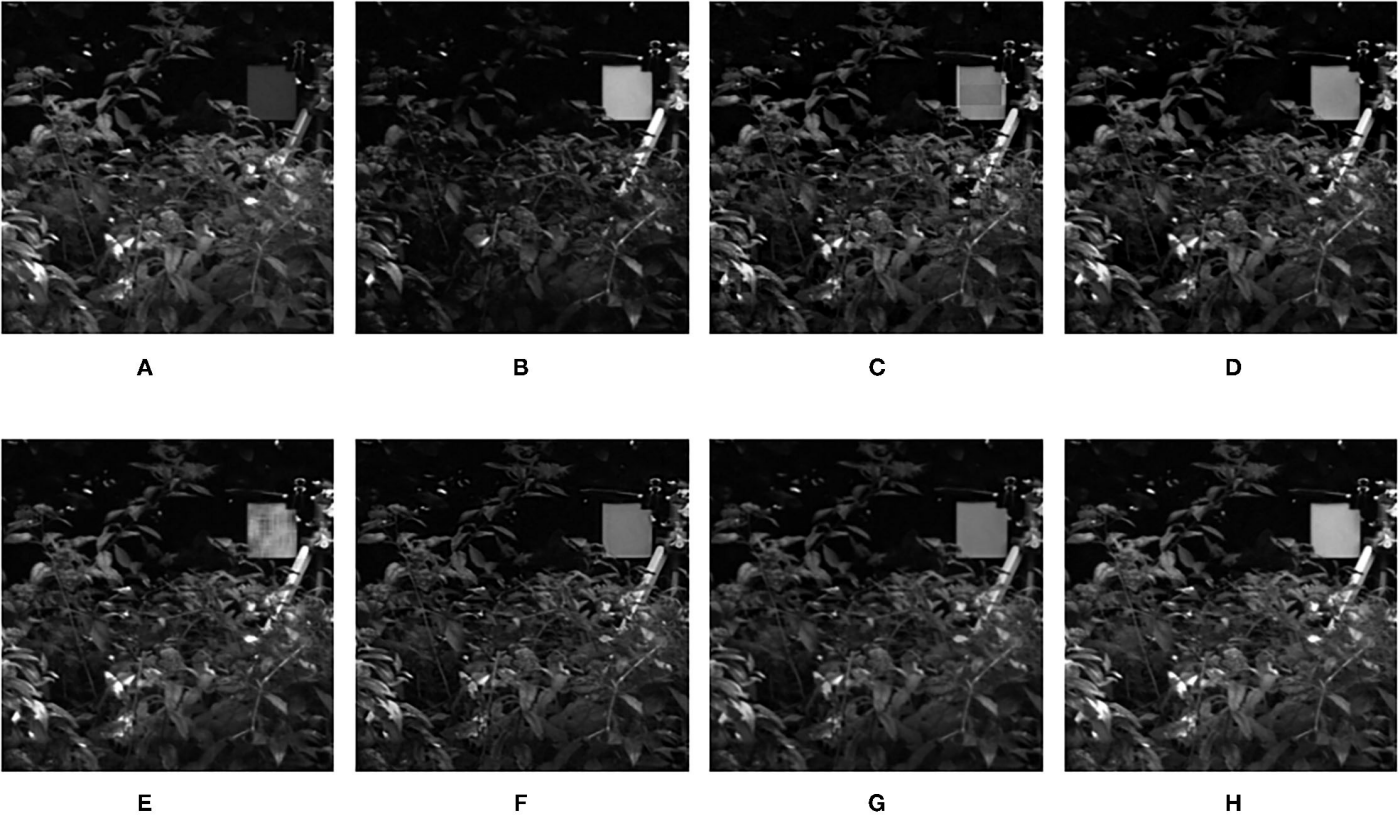
Comparison experimental results of infrared and visible images. **(A)** original image (infrared image); **(B)** original image (visible image); **(C)** DWT; **(D)** LP; **(E)** SR; **(F)** DTCWT-SR; **(G)** VGG; **(H)** MPS.

**Figure 9:**
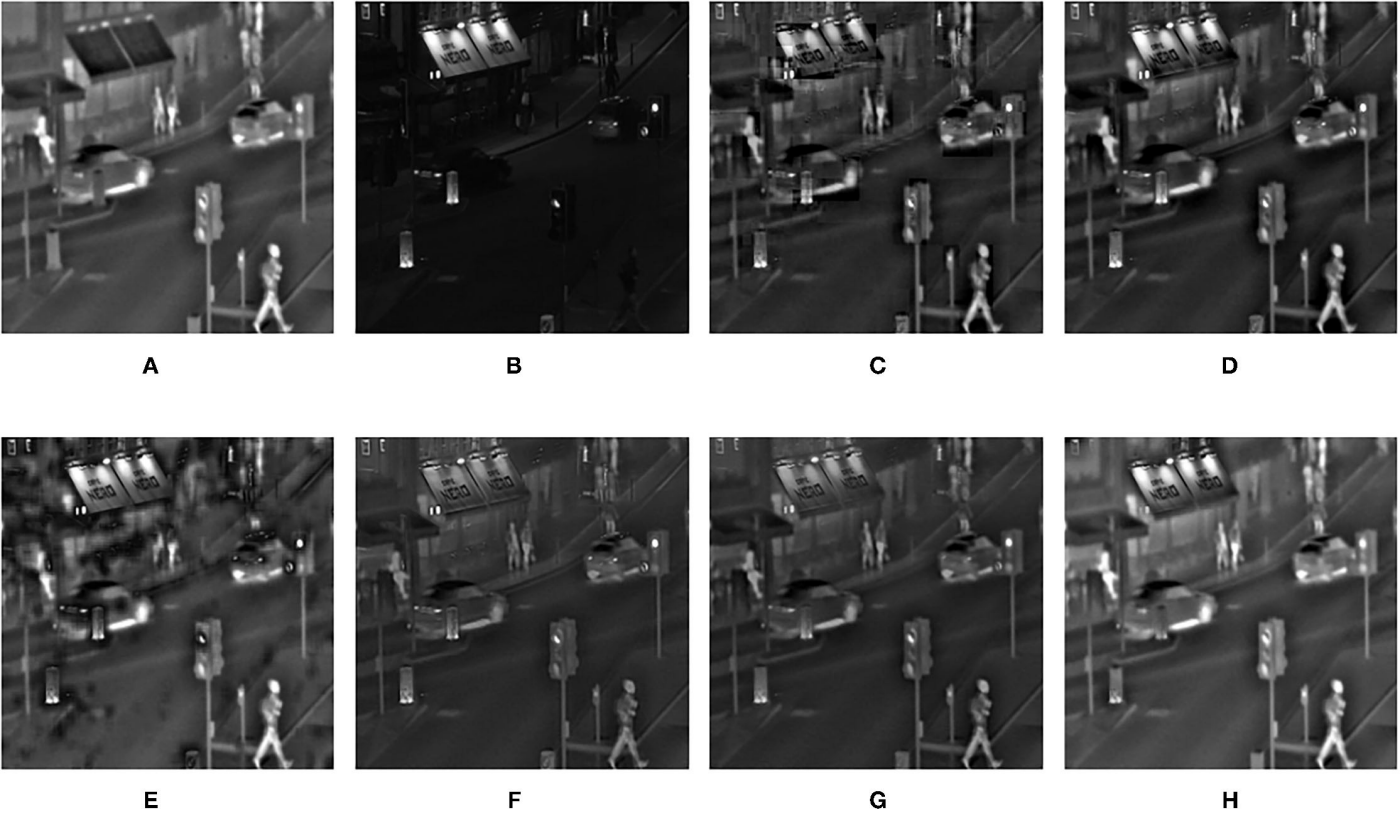
Comparison experimental results of infrared and visible images. **(A)** original image (infrared image); **(B)** original image (visible image); **(C)** DWT; **(D)** LP; **(E)** SR; **(F)** DTCWT-SR; **(G)** VGG; **(H)** MPS.

**Figure 10:**
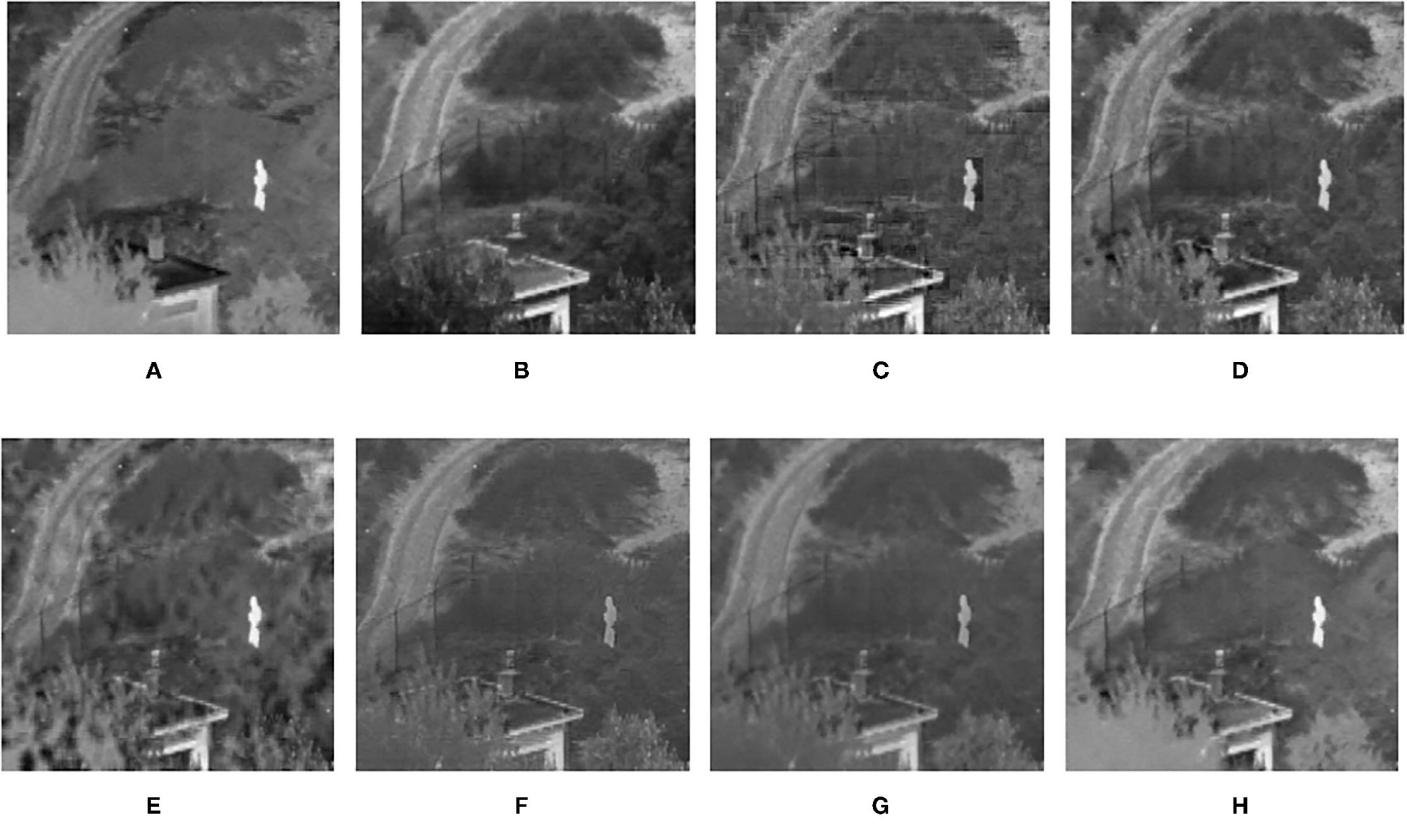
Comparison experimental results of infrared and visible images. **(A)** original image (infrared image); **(B)** original image (visible image); **(C)** DWT; **(D)** LP; **(E)** SR; **(F)** DTCWT-SR; **(G)** VGG; **(H)** MPS.

**Figure 11:**
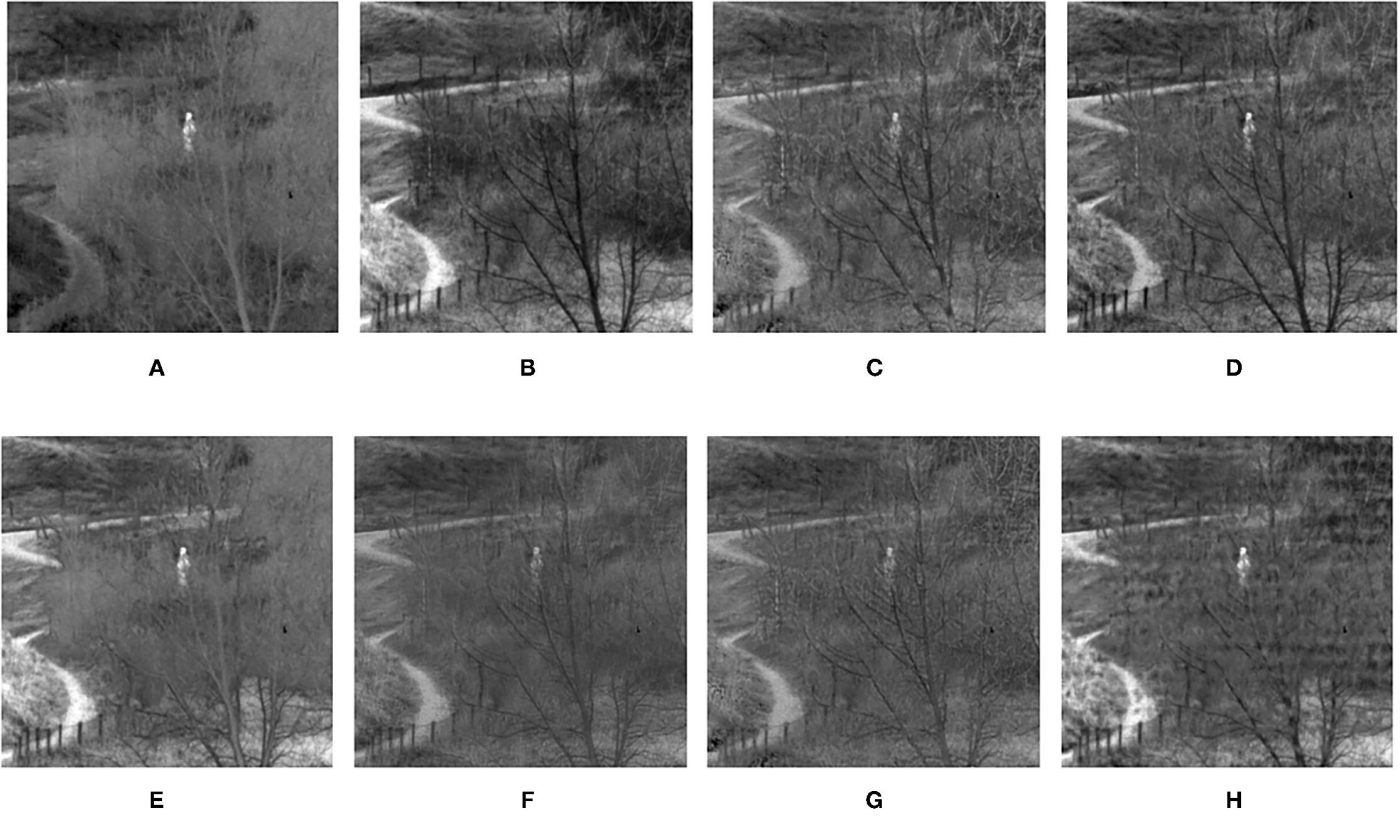
Comparison experimental results of infrared and visible images. **(A)** original image (infrared image); **(B)** original image (visible image); **(C)** DWT; **(D)** LP; **(E)** SR; **(F)** DTCWT-SR; **(G)** VGG; **(H)** MPS.

**Figure 12:**
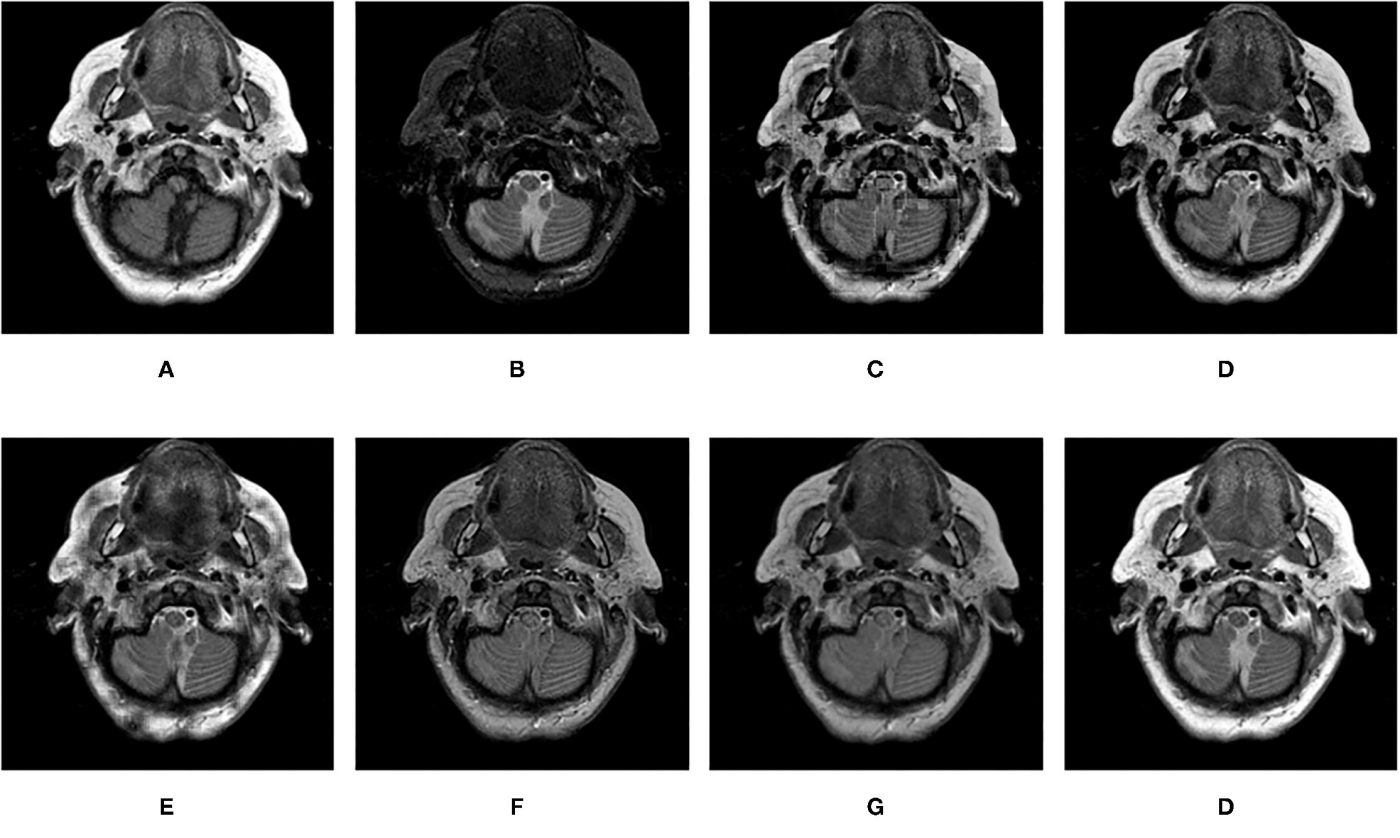
Comparison experimental results of CT and MRI images. **(A)** original image (CT); **(B)** original image (MRI); **(C)** DWT; **(D)** LP; **(E)** SR; **(F)** DTCWT-SR; **(G)** VGG; **(H)** MPS.

**Figure 13:**
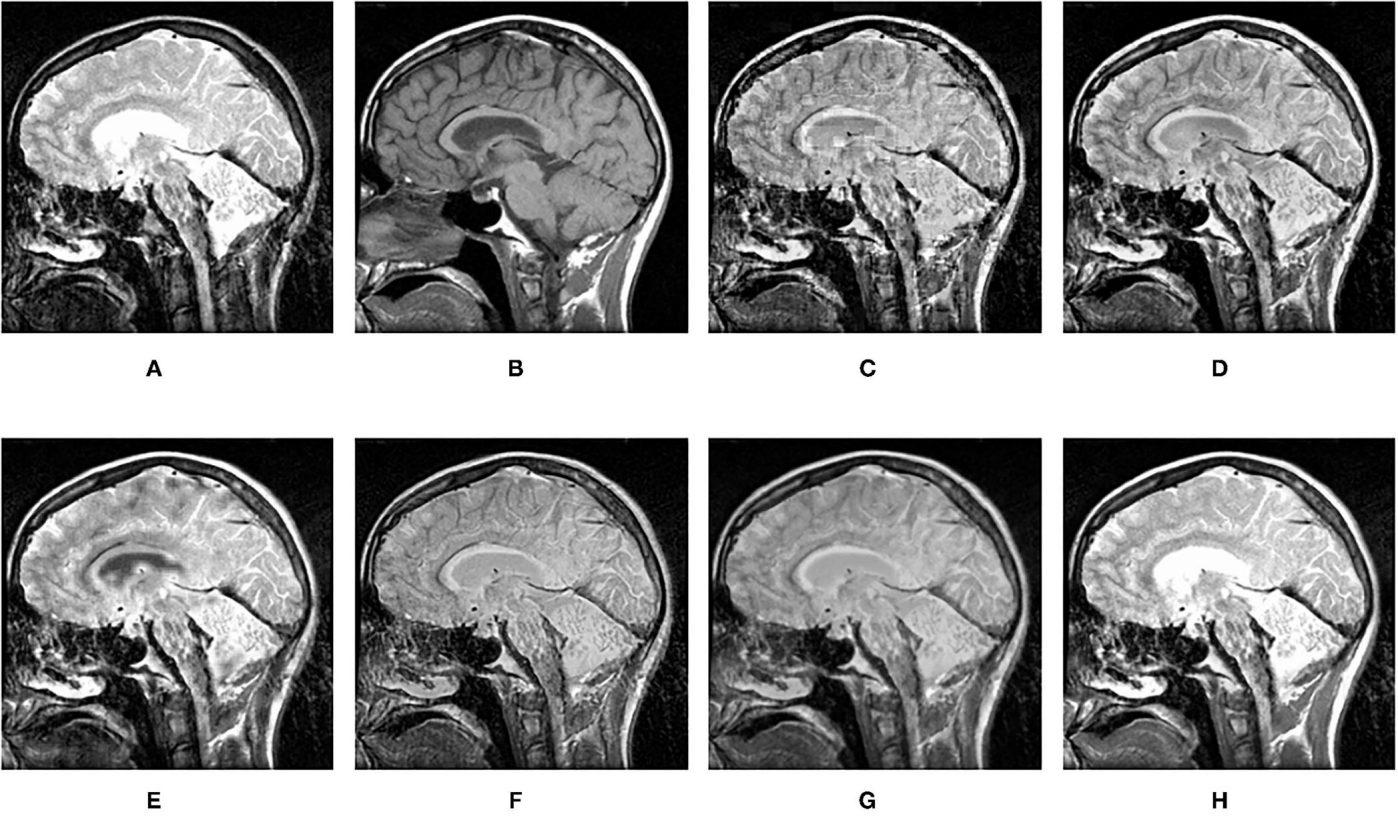
Comparison experimental results of CT and MRI images. **(A)** original image (CT); **(B)** original image (MRI); **(C)** DWT; **(D)** LP; **(E)** SR; **(F)** DTCWT-SR; **(G)** VGG; **(H)** MPS.

**Figure 14:**
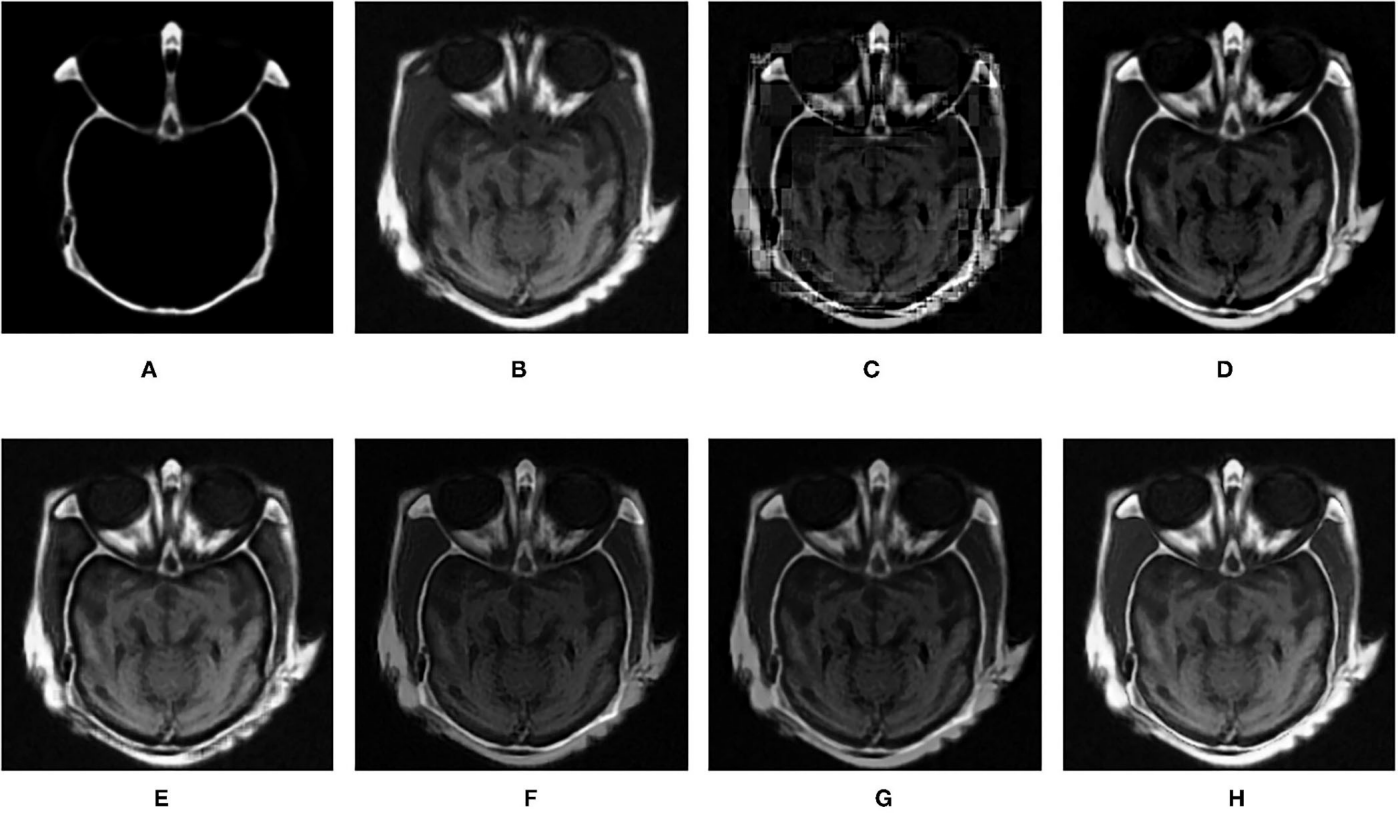
Comparison experimental results of CT and MRI images. **(A)** original image (CT); **(B)** original image (MRI); **(C)** DWT; **(D)** LP; **(E)** SR; **(F)** DTCWT-SR; **(G)** VGG; **(H)** MPS.

**Figure 15:**
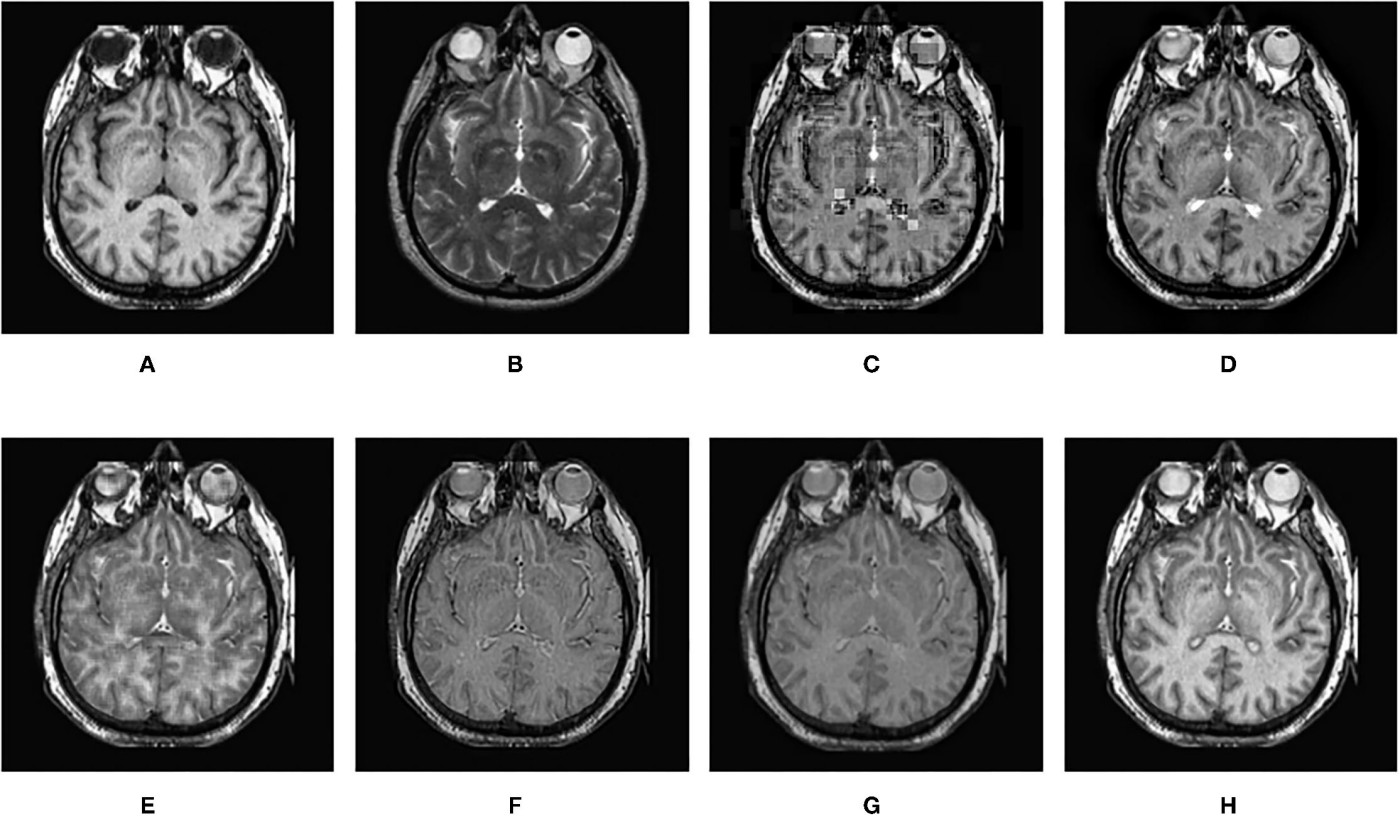
Comparison experimental results of CT and MRI images. **(A)** original image (CT); **(B)** original image (MRI); **(C)** DWT; **(D)** LP; **(E)** SR; **(F)** DTCWT-SR; **(G)** VGG; **(H)** MPS.

In this section, the effectiveness of the proposed method is further verified by comparing the experimental results of this algorithm with other fusion methods. The comparison methods used are DWT (Haribabu and Bindu, 2017) and LP (Burt and Adelson, 1983), SR-based methods (Liu et al., 2016), VGG-Net (Hui et al., 2018), and DTCWT-SR (Singh et al., 2012). In addition to the infrared and visible images used in the previous section, CT and MRI medical images are also used for contrast experiments. The performance of each algorithm is evaluated by calculating the evaluation metrics based on the fusion results. In the experiment, all the experimental source image size is 256 × 256, the fixed patch size is 16 × 16, the step size is 1, and the shrinkage factor k is 200. The proposed method and several comparison algorithms are applied to nine pairs of source images. The experimental results are shown in Figures 7–**15**, respectively. The objective evaluation metrics values of the nine pairs of images are shown in Tables 4, 5.

**Table 4:** Comparison of objective evaluation metrics of infrared and visible images.

**Figure**	**Metrics**	**DWT**	**LP**	**SR**	**DTCWT-SR**	**VGG**	**MPS**
	*SD*	57.3495	59.1760	55.0023	65.0175	49.1788	**65.0244**
	*MI*	0.3915	0.3981	0.7197	0.5625	0.4373	**0.8811**
Figures 7A,B	*SSIM*	0.6308	0.6481	0.6602	0.6025	0.6266	**0.6699**
	*Q*_*G*_	0.6573	0.6990	**0.7014**	0.6874	0.5975	0.6813
	*Q*_*P*_	0.6769	0.7765	0.7392	0.7586	0.7139	**0.7916**
	*SD*	37.3080	39.6529	38.5454	41.7521	32.5971	**42.1726**
	*MI*	0.5262	0.5779	0.6457	0.5969	0.6614	**0.9720**
Figures 8A,B	*SSIM*	0.7674	0.7688	**0.8170**	0.8013	0.7603	0.8017
	*Q*_*G*_	0.5558	0.6168	0.5753	0.6094	0.5995	**0.6748**
	*Q*_*P*_	0.6769	0.7764	0.6895	0.7632	0.7659	**0.8349**
	*SD*	31.6360	35.0567	35.3218	**36.0795**	23.3351	35.0392
	*MI*	0.3357	0.4055	0.4509	0.3861	0.4190	**0.9292**
Figures 9A,B	*SSIM*	0.5701	0.6088	0.5350	0.4449	0.4284	**0.6157**
	*Q*_*G*_	0.5755	0.6560	0.4487	0.5945	0.5352	**0.7249**
	*Q*_*P*_	0.4050	0.5384	0.2579	0.5002	0.5816	**0.7004**
	*SD*	28.3593	29.2224	28.5693	29.7191	22.83747	**29.8865**
	*MI*	0.2238	0.2356	0.2720	0.2582	0.2604	**0.7066**
Figures 10A,B	*SSIM*	0.6288	**0.7088**	0.6331	0.4864	0.5494	0.6926
	*Q*_*G*_	0.3999	0.4835	0.3304	0.4211	0.3482	**0.5204**
	*Q*_*P*_	0.1892	0.2996	0.1220	0.2577	0.2541	**0.3927**
	*SD*	23.9236	25.6275	31.2000	**39.5077**	29.1652	31.7625
	*MI*	0.1528	0.1573	0.2883	0.4184	0.3954	**0.6704**
Figures 11A,B	*SSIM*	0.4223	0.4544	0.4624	0.4025	0.4351	**0.4911**
	*Q*_*G*_	0.4217	0.5184	0.3987	0.5021	0.3749	**0.5204**
	*Q*_*P*_	0.2256	0.3745	0.1827	0.3612	0.2683	**0.4304**

*Bold values mean maximum value of the same metrices in the same group of comparative experiments*.

**Table 5:** Comparison of objective evaluation metrics of CT and MRI images.

**Figure**	**Metrics**	**DWT**	**LP**	**SR**	**DTCWT-SR**	**VGG**	**MPS**
	*SD*	56.2694	60.5508	59.2485	72.1939	49.4054	**73.6843**
	*MI*	0.6266	0.6713	0.7032	0.7449	0.6849	**1.0761**
Figures 12A,B	*SSIM*	0.6846	0.7114	0.7088	0.6631	0.5067	**0.7318**
	*Q*_*G*_	0.6618	0.6706	0.6818	0.6728	0.3410	**0.7696**
	*Q*_*P*_	0.3756	0.4625	0.2952	0.3945	0.5255	**0.6941**
	*SD*	75.3185	77.7486	80.6627	82.7177	68.0464	**86.4508**
	*MI*	0.4175	0.4496	0.6142	0.4336	0.5063	**0.7824**
Figures 13A,B	*SSIM*	0.5358	0.5861	0.5921	0.5969	0.5787	**0.6041**
	*Q*_*G*_	0.4343	0.5262	0.5425	0.4216	0.4322	**0.5806**
	*Q*_*P*_	0.2928	0.4069	0.3859	0.3193	0.3986	**0.5129**
	*SD*	45.5362	53.7899	55.2056	53.5713	37.3261	**59.2069**
	*MI*	0.4510	0.4551	0.8655	0.3547	0.6078	**1.1221**
Figures 14A,B	*SSIM*	0.3849	0.4403	0.4937	0.5016	0.3702	**0.5057**
	*Q*_*G*_	0.6453	0.6430	0.8465	0.8056	0.6750	**0.9191**
	*Q*_*P*_	0.2833	0.5291	0.5418	0.5613	0.5755	**0.5769**
	*SD*	62.0558	66.6555	65.3679	69.7711	55.0330	**72.7695**
	*MI*	0.6098	0.6060	0.7530	0.4336	0.6993	**0.9532**
Figures 15A,B	*SSIM*	0.5962	0.6136	0.6569	0.6410	0.5476	**0.6756**
	*Q*_*G*_	0.5955	0.5743	0.6552	0.3626	0.3392	**0.7100**
	*Q*_*P*_	0.2531	0.4005	0.3128	0.2664	0.3675	**0.6454**

*Bold values mean maximum value of the same metrices in the same group of comparative experiments*.

It can be seen from the table that in most cases, the algorithm proposed in this article can achieve optimal results, especially for CT and MRI images, the various metrics of the results of MPS are much higher than other methods. For infrared and visible images, the method in this article can also achieve optimal results under more than half of the evaluation metrics. These results show that the proposed method is better than other methods for multi-modal image fusion. This advantage mainly benefits from two aspects: (i) The sliding window method is adopted to divide the image into several sub-images, so the local information of the image can be captured well; (ii) MPS method is an accurate decomposition and reconstruction method, so in the process of image fusion, there will be no loss of information due to the solution.

Further analysis of the experimental results shows that: (i) On the whole, VGG-Net has the worst performance in all cases. Compared with other comparison methods, there is a big gap in various evaluation metrics. This is because the information captured is insufficient in the layer-by-layer feature extraction of the source image, and when the details of the fusion image are weighted by the final weight graph, the contrast of the initial detail part of the fusion image is reduced; (ii) Among the two multi-scale methods used, DWT fusion method performs poorly. This is because the DWT method is based on Haar wavelet to achieve fusion, which can only capture image features in horizontal and vertical directions but cannot capture more basic features of the image; LP method is better than the DWT method because the Laplacian pyramid generates only one band-pass component at each scale, which reduces the possibility of being affected by noise; (iii) The results obtained by SR method are better than other multi-scale methods in most cases but not as good as the proposed method. This is because the signal representation ability of SR is better than that of multi-scale transformation, and errors will occur in the process of signal reconstruction, which is unavoidable for the SR method. The method proposed in this article can effectively avoid this problem by non-destructive tensor reconstruction. In addition, the “max-L1” rule of direct fusion in the spatial domain will lead to spatial inconsistency, which affects the performance of the SR method; (iv) DTCWT-SR is an method that multi-scale method combined with SR method. By comparing the objective evaluation metrics, the fusion performance of the algorithm is better than SR in some aspects, but it is still poor compared with MPS.

In addition to objective evaluation, the performance of the algorithm in this article is also discussed through some visual comparisons of the fused images. In general, the proposed method achieves the best visual effect among all the fusion images.

The fusion results of infrared-visible images are shown in Figures 7–11. It can be seen from the figure that the method proposed in this article has good adaptability, and the fusion images are obtained to retain the information of the infrared and visible images, respectively. In Figure 7, both the multi-scale fusion method and SR show varying degrees of artificial traces at the junction between the trees and the sky in the upper left corner, while DTCWT-SR and VGG-Net resulted in severe contrast loss. In Figure 8, the white squares in infrared picture are dimming in varying degrees in DWT, LP, SR, DTCWT-SR, and VGG-Net methods, and the leaf luster in the visible image is not well-displayed in the VGG-Net method. In Figure 9, DWT and SR show the phenomenon of information loss. LP, DTCWT-SR, and VGG can get relatively complete fusion images, but the brightness is weaker than MPS. The clarity of the billboard in the upper left corner of the fused image is better in the MPS method. In Figure 10, the fused images obtained by DWT and SR show some small black blocks, that is information loss, while the human shape brightness on the right side of the images obtained by LP, DTCWT-SR, and VGG method is low. The reason for these shortcomings is the fusion rules used in the fusion process all have a certain degree of weighting on the source image. Our fusion rules based on the sigmoid function can well avoid these shortcomings, that is, in the image, whose colors are only black and white, the weight of the white part of the image will be much larger than that of the black part, thus, evolving into the Choose-max rule. In Figure 11, compared with the other five comparison methods, it can be seen that the human figure on the right and the branch on the lower right corner of the fusion image obtained by MPS have the highest resolution.

Figures 12–15 are the fusion results of CT and MRI medical images. It can be seen from the experimental results that the DWT method cannot to be applied to the fusion of medical images, and the other four methods can obtain a complete image. In Figure 12, LP, DTCWT-SR, and VGG-Net methods have no loss in details, but the sharpness of the light and dark junction is insufficient, the edge is blurred, and the contrast is lost. However, the bottom of the fused image obtained by the SR method is fractured, indicating that there is information loss. In Figure 13, the spine in the lower right corner and the jaw in the lower left corner of the image obtained by MPS were more clear than the other five methods, the brain vein was also clearer, and the contrast was higher than the other five methods. In Figure 14, the fused images obtained by LP and SR methods were fractured at the lower right corner. Although DTCWT-SR and VGG methods obtained relatively complete fusion images, there is a certain degree of contrast loss. In Figure 15, LP, DTCWT-SR, and VGG-Net methods have some contrast loss, especially in the middle part, at the same time, the image obtained by the SR method presents spatial dislocation at both sides of the eyeball and a certain degree of distortion appears at the position of white connection of the two images. The SR method also has similar shortcomings in this regard, please refer to the lower right corner of the image.

## 5. Conclusion

In this article, we propose a method based on MPS for multi-modal image fusion. First, the source images are initialized into a three-dimensional tensor, and then the tensor is decomposed into several sub-tensors by using a sliding window to obtain the corresponding features. The core matrix is fused by the fusion rule based on the sigmoid function, and the fused image is obtained by multiplying the left-right factor matrix. In this article, we use a sliding window to avoid blocking effects, and fully consider the local information of the source images by dividing the source image into a set of sub-images. The experimental results show that the proposed algorithm is feasible and effective for image fusion. Being different from the average fusion rule of the multi-scale method and the “Max-L1” fusion rule of the SR method, the fusion rule based on the sigmoid function used in the article is more effective, but it also makes the fusion process more complicated of the proposed method. Future study will focus on further exploring a more effective fusion rule to improve the fusion results.

## Data Availability Statement

The original contributions presented in the study are included in the article/supplementary material, further inquiries can be directed to the corresponding author/s.

## Author Contributions

YL designed the algorithm. RW performed experiments and wrote this article. QG, DS, and DZ revised the manuscript. All authors confirmed the submitted version.

## Funding

This work was supported by Nature Science Foundation of Anhui (2008085MF183), The Key Science Program of Anhui Education Department (KJ2018A0012), The Research Fund for Doctor of Anhui University (J01003266), and National Natural Science Foundation of China (NSFC)(61402004).

## Conflict of Interest

The authors declare that the research was conducted in the absence of any commercial or financial relationships that could be construed as a potential conflict of interest.

## Publisher's Note

All claims expressed in this article are solely those of the authors and do not necessarily represent those of their affiliated organizations, or those of the publisher, the editors and the reviewers. Any product that may be evaluated in this article, or claim that may be made by its manufacturer, is not guaranteed or endorsed by the publisher.
